# Probiotic *Gordonia alkanivorans* Enhances Phagocytic Function of Porcine Alveolar Macrophages and Modulates Immune Responses in Piglets

**DOI:** 10.3390/vetsci13030271

**Published:** 2026-03-15

**Authors:** Xuwen Lu, Jieyang Wu, Zhiyi Zhang, Xiang Li, Zikui Liu, Gaofeng Liu, Guiping Wang

**Affiliations:** 1 College of Veterinary Medicine, Hunan Agricultural University, Changsha 410128, China; 2 Hunan Canzoho Biological Technology Co., Ltd., Changsha 410000, China; 3 Academic Affairs Research Office, Shaoyang Industry Polytechnic College, Shaoyang 422000, China

**Keywords:** *G. alkanivorans*, pig alveolar macrophages, PRRSV, intestinal microorganisms

## Abstract

This study aimed to evaluate effect of *Gordonia alkanivorans* on phagocytic activity of porcine alveolar macrophages (PAMs) and immune function in piglets. Quantitative PCR and fluorescence tracing were used to measure phagocytic efficiency of *G. alkanivorans*-intervened PAMs against PRRSV and *E. coli*. Sixty-four 45-day-old cross-bred piglets with equal sex were randomly divided into four groups (*n* = 16/group). Growth performance, immune function, and intestinal flora were analyzed. *G. alkanivorans* extract exhibited half cytotoxic concentration of 36.43 mg/mL, half effective concentration of 0.1009 mg/mL, and half inhibitory concentration of 0.0043 mg/mL in PAMs, significantly increasing their phagocytic efficiency by 98.5% against PRRSV and 2.31- to 13.46-fold against *E. coli*. Dietary supplementation with *G. alkanivorans* elevated antibody-positive rates against classical swine fever virus (47.92%) and pseudorabies virus (14.58%), modified serum cytokine: Interleukin (IL)-1β, IL-2, Tumor Necrosis Factor -α, Interferon (IFN)-α, IFN-γ, IL-4, and IL-10 (−144.51% to +191.72%). It increased intestinal operational taxonomic units by 152%, the Shannon index by 14.62%, and the Chao index by 11.37%, while reducing the Firmicutes/Bacteroidetes ratio by 713.90%. In conclusion, *G. alkanivorans* enhances immunity and antiviral activity in piglets by gut and immune regulation.

## Simple Summary

As a probiotic strain rich in glycolipid, *Gordonia alkanivorans* (*G. alkanivorans*) exhibits significant potential for enhancing animal immunity. Using in vitro porcine alveolar macrophage (PAM) models and in vivo animal experiments, this study demonstrated that *G. alkanivorans* significantly enhanced the phagocytic capacity of PAMs against porcine reproductive and respiratory syndrome virus (PRRSV) and *Escherichia coli (E. coli)*. Furthermore, it increased the positive rates of virus-specific antibody in piglets, modulated serum cytokine profiles, and improved intestinal microbial diversity. Collectively, *G. alkanivorans* exerts potent antiviral activity and improves piglet health by the enhancement of immune function and modulation of intestinal microecology.

## 1. Introduction

Antibiotics play a crucial role in the control of infectious diseases in pigs. However, excessive antibiotics use not promotes the widespread emergence of drug resistant strains but also threatens human health via the food chain [1]. In line with policies aimed at reducing and banning antibiotic growth promoters, microecological preparations have attracted considerable attention as green and sustainable alternative for disease control and growth promotion in livestock husbandry [2]. In China, numerous probiotic products for livestock and poultry feed and drinking water are currently under research, covering various types of strains and application scenarios. These preparations can enhance animal health through multiple mechanisms, including competitive exclusion [3], immunomodulation [4], and regulation of intestinal microbial homeostasis [5]. Nevertheless, their practical application remains limited. First, probiotic strains are scarce [6]. Currently, probiotics approved by the Ministry of Agriculture and Rural Affairs of China are mainly restricted to *Lactobacillus* and *Bacillus*, representing only a small fraction of the total microbial diversity [7]. In China, only approximately 35 probiotics species are permitted for animal feed, accounting for less than 5% of the agricultural microbial strains preserved in the national microbial resource bank [8]. Furthermore, the industrial conversion rate of patented microbial strains in China is only 3.3%, and fewer than 10% of native strains have achieved large-scale industrial application indicating that most strains remain limited to laboratory research [8]. Second, the colonization efficiency of conventional probiotics is low. Most struggle to tolerate the gastric environment, with survival rates generally below 20%, which severely restricts their intestinal colonization and functional expression [9]. Therefore, the development of novel probiotic preparations with strong tolerance holds great potential for improving application effect in livestock production [10].

*Gordonia alkanivorans* (*G. alkanivorans*), belongs to the phylum Actinomycetes, along with *Mycobacterium*. Previous studies have reported that the cell walls of these bacteria are rich in glycolipid components, whereas *G. alkanivorans* has been shown to exert antibacterial activity [11]. Its unique structure may enhance the host innate immune response by activating pattern recognition receptors [12]. Additionally, its extreme tolerance, including resistance to pH 2.0–10.0 and tolerance to 0–15% salinity, suggesting a strong colonization in the digestive tract (typically pH 3.0–8.0 and 0–5% salinity) [13]. Notably, research on *G. alkanivorans* have focused primarily on its application in petroleum pollutant degradation [14], while its immune regulatory function in livestock production remains largely unexplored.

Porcine alveolar macrophage (PAM) serve as the core component of the innate immune defense in the porcine respiratory tract and mediating Pathogen clearance through multiple synergistic mechanisms: (1) Pathogen recognition. Pattern recognition receptors expressed on the PAM surface directly bind to pathogen associated molecular patterns, triggering rapid activation of innate immune responses [15]. (2) Phagocytosis and clearance. PAMs efficiently engulf pathogens, followed by degradation of internalized microbes via lysosomal enzymes and reactive oxygen species [16]. (3) Inflammatory amplification. Secretion of proinflammatory cytokines and chemokines amplifies inflammatory signaling cascades to recruit additional immune cells [17]. (4) Immune homeostasis regulation. Release of antiinflammatory cytokines modulates the intensity of inflammation, preventing excessive tissue damage [18]. However, porcine reproductive and PRRSV impairs PAM function through immune escape strategies [19], including the inhibition of interferon signaling and the induction of abnormal autophagy [20], thereby increasing the risk of immunosuppression and secondary infections. Based on the reports of antibacterial activity of *G. alkanivorans* [11], this study explores its role on PAMs’ resistance to PRRSV infection for the first time and evaluates its potential as an anti-PRRSV immunostimulant.

As a virus with high host specificity and cell tropism, PRRSV’s targeting of PAMs is a consensus conclusion in the field of research on the virus’s pathogenic mechanism. From the perspective of viral invasion mechanism, PRRSV mainly binds to specific receptors on the surface of PAMs through its surface glycoproteins, including CD163 and sialoadhesin [21], thereby completing the adsorption and internalization of viral particles [22]. From the perspective of viral replication characteristics, after PRRSV enters PAMs, it utilizes organelles of the host cell to complete the replication of the viral genome, the synthesis and assembly of structural proteins, and then releases progeny viruses through budding. This process impairs key functional targets of PAMs (phagocytic capacity and immune signaling) and induces cell apoptosis [23], ultimately leading to the suppression of the host’s innate and adaptive immune responses [24].

Accordingly, this study established an in vitro in vivo dual verification system, a classic and reliable research model in probiotic and antiviral research [25]. The objectives were to: (1) clarify the inhibitory effect and mechanism of *G. alkanivorans* and its cell wall components on PRRSV infection in PAMs at the cellular level; (2) evaluate the impacts of *G. alkanivorans* supplementation on growth performance, humoral immunity, and intestinal flora of weaned piglets in vivo; (3) explore its potential as a novel probiotic to enhance piglet immune function and resistance to PRRSV infection. Using PAMs as a model, we analyzed the inhibitory effects of *G. alkanivorans* and its cell wall components on PRRSV infection. By examining the dose–response relationships between this strain and growth performance, antibody titers, and intestinal flora, we evaluated its effects on production performance, immune function, and intestinal health. The study provides theoretical and experimental support for the development and application of novel probiotics and offers a promising candidate strain for the green prevention and control of viral diseases in pigs.

## 2. Materials and Methods

### 2.1. Animal Ethics

This study was approved by the Biomedical Research Ethics Committee of Hunan Agricultural University (approval number: 2024-140) and conducted in strict compliance with the ARRIVE guidelines and relevant national regulation on animal welfare and experimental ethics. All piglets used in this study were provided by a commercial pig farm with informed consent from the owner. Piglets were housed under standard environmental conditions with free access to feed and water. Sample sizes were determined in adherence to the 3Rs principle (Replacement, Reduction, Refinement).

### 2.2. Cells and Microorganism

The commercial PAM cell line 3D4/21 was purchased from Saiye (Suzhou) Biotechnology Co., Ltd. (Suzhou, China) and stored in our laboratory. No primary PAM were isolated from live animals, and no animal euthanasia was involved in the experiments. The highly pathogenic PRRSV JXA1 strain was kindly provided by Professor Meng Ge of Hunan Agricultural University. It was originally isolated from lung tissue of a PRRS-positive piglet on a commercial farm in Shaoyang, Hunan, China. The virus was serially passaged 6 times in MARC-145 cells and its infectious titer was determined to be 10^6.0^ TCID_50_/mL. The pathogenic *E. coli* strain XT was isolated from diarrheic weaned piglets (21–28 day old) on a large-scale pig farm in Xiangtan City, Hunan, China. It was aerobically cultured on LB agar at 37 °C for 24 h, serially passed six generations for phenotypic stability, and preserved as 30% (*v*/*v*) glycerol-LB stocks at −80 °C in our laboratory for subsequent use. *G. alkanivorans* strain HN (China General Microbiological Culture Collection Center, No. 26361) and its cell wall extract were kindly provided by Hunan Canon Zhenghe Biotechnology Co., Ltd. (Canon Zhenghe, Changsha, China), which also supplied the recommended dosages (10 mg/mL for the strain, 6.25 mg/mL for the cell wall extract) used throughout this study. The cell wall extracts of *G. alkanivorans* were prepared according to a modified method described previously [26]. Briefly, *G. alkanivorans* cells were cultured and harvested by centrifugation, and washed with Phosphate-Buffered Saline (PBS) (pH 7.4) to remove residual medium. The cell pellets were resuspended and disrupted ultrasonic on ice to ensure complete lysis. After lysis, unbroken cells and cell debris were removed by centrifugation. The crude cell wall fractions was washed with PBS, treated with 2% (*w*/*v*) sodium dodecyl sulfate at 37 °C for 1 h to remove membrane proteins, and washed repeatedly by centrifugation until no protein was detected by the BCA protein assay kit. Finally, the purified cell wall extracts were lyophilized and stored at −80 °C until use.

### 2.3. Determination Both Drug Toxicity and Antiviral Effects

Drug toxicity was evaluated with safe drug concentrations and half cytotoxic concentration (CC_50_). Antiviral activity was evaluated with half effective concentration (EC_50_) and half inhibitory concentration (IC_50_).

Determination of safe drug concentrations: PAMs were seeded in 96-well plates at a density of 1 × 10^5^ cells/well and incubated for 2 h. The culture medium was then discarded, *G. alkanivorans* at concentrations of 0 (control), 0.001, 0.01, 0.1, 1.0, 10, and 100 mg/mL (total 7 groups), and its cell wall extracts at concentrations of 0 (control), 0.098, 0.39, 1.56, 6.25, 25, and 100 µg/mL (total 7 groups) were added to the respective wells with *n* = 3 wells per group. After 24 h of incubation, cytopathic effects were observed under a biological microscope (Model LWD200-371, Shanghai Cwell Optoelectronic Technology Co., Ltd., Shanghai, China) and photographed. The drug concentration that causes no significant difference in cell morphology, compared with the control group of PAMs (without *G. alkanivorans* addition), is defined as the safe concentration.

Determination of the CC_50_: PAMs 3d4/21 were seeded at 1 × 10^5^ cells/well in a 96-well plate and incubated for 2 h, then the culture medium was discarded. *G. alkanivorans* at concentrations of 0 (control), 0.001, 0.01, 0.1, 1.0, 10, 100 mg/mL were separately added into these wells for 24 h, with no PRRSV inoculated during this process. A 10% CCK-8 reagent was added into each well and incubated for 2 h. The optical density (OD) values were measured with a microplate reader (TECAN F50) at 450 nm, and the data were fit to a curve using GraphPad Prism 6 version (GraphPad Software, San Diego, CA, USA) fitting curve. The CC_50_ was defined as the concentration of the reagent that reduces PAM viability by 50%. Specifically, CC_50_ refers to the concentration of a drug or reagent that causes 50% cellular damage or inhibits the survival of half of the cultured target cells under specific incubation conditions, and it is a core indicator for evaluating the cytotoxicity of drugs/reagents. A higher CC_50_ value indicates lower cytotoxicity of the drug or reagent to the target cells.

Determination of the EC_50_: Based on the results of the cytotoxicity assay, PAMs were treated with *G. alkanivorans* at concentrations of 0 (control), 0.001, 0.01, 0.1, 1.0, and 10 mg/mL, followed by inoculation with PRRSV at a multiplicity of infection corresponding to 100 TCID_50_ (50% Tissue Culture Infectious Dose) for 48 h. The 24 h incubation for CC_50_ was intended to evaluate the maximal concentration of the strain on PAMs, whereas the extended 48 h incubation for EC_50_ was used to ensure sufficient time for PRRSV replication and the expression of the antiviral effect of *G. alkanivorans*. Subsequently, cells were incubated with CCK-8 reagent for 2 h, after which OD_450_ values were determined and curve was fitted. The half maximal effective concentration (EC_50_) was defined as the concentration that protects 50% of PAM viability during PRRSV challenge. EC_50_ refers to the concentration of a drug, reagent, or exogenous substance that produces 50% of the maximum biological response in target cells, tissues, or organisms under specific defined experimental conditions. It is a core indicator for evaluating biological activity, with a lower EC_50_ representing higher antiviral activity potency at lower concentration.

Determination of the IC_50_: Based on the results of the safe conc cytotoxicity assay, which identified 10 mg/mL as the maximal non cytotoxic of *G. alkanivorans* for PAMs, cells were treated with *G. alkanivorans* at concentrations of 0 (control), 0.001, 0.01, 0.1, 1.0, and 10 mg/mL, followed by inoculation with PRRSV at 100 TCID_50_ per well for 48 h. Subsequently, PAM lysates were collected, and viral copy numbers were quantified by a Quantitative PCR (qPCR) using a commercial qPCR kit (Hunan Guanmu Biotechnology Co., Ltd., Cat. No. 2024021, Changsha, China). The viral proliferation inhibition rate was calculated using the Comparative Threshold Cycle (ΔΔCt) method, and GraphPad Prism 6 was used to fit the curve for determining the half-maximal inhibitory concentration (IC_50_), which was defined as the concentration of the strain that inhibits PRRSV replication by 50%. IC_50_ refers to the concentration of a drug, reagent, or exogenous substance that achieves 50% inhibition of a specific biological process in target cells or tissues under defined experimental conditions. It is a key indicator for evaluating the inhibitory potency of a substance, with a lower IC_50_ value indicating a stronger inhibitory effect.

### 2.4. Phagocytosis Efficiency of PAMs Treated with G. alkanivorans Cell-Wall Extract

Fluorescence tracing assays and qPCR were performed to evaluate effect of *G. alkanivorans* cell wall extract on the phagocytic efficiency of PAMs against PRRSV, while colony-forming unit (CFU) counting was used to determine its impact on the phagocytic efficiency of PAMs against *E. coli*. PAM phagocytic efficiency was designated as the primary outcome measure.

Fluorescence tracing assay: 100 µL of DiD fluorescent dye (initial concentration: 1 mM) was mixed with 1.0 mL of PRRSV (100 TCID_50_), resulting in a final working concentration of 90.9 μM for dye. The mixture was oscillated at 37 °C in the dark for 3 h. Free dye was then removed using a NAP-5 adsorption column; after elution with PBS buffer, the mixture was filtered through a 0.22 µm filter membrane, aliquoted, and stored at −80 °C. Based on the cytotoxicity assay results of the *G. alkanivorans* cell wall extract, PAMs were pretreated with the cell wall extract at concentrations of 0 (control), 0.1, 1.0, 10 µg/mL for 4 h, respectively. Subsequently, the PAMs were incubated with DiD-labeled PRRSV for 6 h to facilitate phagocytosis [27]. Specifically, the following steps were performed sequentially: fixation with 4% paraformaldehyde (Biosharp, BL539A), membrane permeabilization with 0.2% Triton X-100 (Beijing Solaibao Technology Co., Ltd., T8200, Beijing, China), blocking with 1% Bovine Serum Albumin, staining with 1% β-actin antibody (Wuhan Sanying Biotechnology Co., Ltd., 21020685, Wuhan, China), and nuclear staining with DAPI (Beijing Solaibao Technology Co., Ltd., No. 202311005). Fluorescence intensity was observed and imaged under a fluorescence microscope (Axio Observer 7, ZEISS, Germany) and quantified u sing Image J 1.8 version [27].

QPCR: Following 6 h of co-incubation of PAMs with PRRSV (to facilitate viral engulfment), the cell monolayer was gently washed three times with pre-warmed PBS (pH 7.4) to remove unengulfed free PRRSV particles adherent to the cell surface. PAMs were then lysed, and total nucleic acid was extracted. Viral copy numbers were quantified using a commercial qPCR kit with a real-time fluorescence quantitative PCR instrument (CFX Connect Real-Time System, Bio-RAD, Hercules, CA, USA), and the viral phagocytosis rate was calculated using the ΔΔCt method [23]. The reagents stored in the kit were used as the control group instead of PRRSV.

CFU counting assay: PAMs were incubated with *E. coli* (1 × 10^8^ CFU/mL) for 2 h. After cell homogenization and lysis, the lysates were plated on tryptic soy agar (TSA) plates, which were then incubated at 37 °C for 24 h before colony counting. TSA agar plates without bacterial inoculation were used as sterility controls.

### 2.5. Animal Experiments

Healthy 45-day-old weaned cross-bred piglets (Duroc × Landrace × Yorkshire, *n* = 64) with a balanced sex ratio (32 males and 32 females) were selected for this study. All piglets were confirmed to be in good health status by the following criteria: no clinical symptoms of infectious diseases, normal appetite and mental status, consistent body weight, and negative for major pathogenic viruses (PRRSV, ASFV, and PCV2) as detected by qPCR prior to the experiment. The initial body weights of piglets in each group were statistically similar: Control 14.41 kg, Low 14.66 kg, Medium 14.27 kg, High 15.05 kg, ensuring baseline homogeneity. According to the immunization protocol, all 64 piglets were injected with Classical Swine Fever (CSFV) vaccine at 22 days of age and Pseudorabies Virus (PRV) vaccine at 30 days of age. Piglets were randomly allocated to four groups via a computer-generated random number table, with 16 piglets per group (8 males, 8 females), divided into 4 replicates of 4 piglets each. The sample size (64 piglets,16 per group) was determined on three considerations: 1. Power analysis (α = 0.05, power = 0.80) and an expected 15% difference in immune indices [28]; 2. Compliance with veterinary guidelines for swine nutrition and immunology [29]; 3. Alignment with the farm’s breeding capacity (4 pens per treatment, 4 piglets per pen) to ensure experimental reproducibility. Three experimental groups received piglet feed supplemented with *G. alkanivorans* at 0.67, 2.0, and 6.0 kg/ton, respectively, while the control group was fed basal diet without *G. alkanivorans* supplementation. These doses were selected based on preliminary farm trials showing the optimal effect at 2.0 kg/ton, which served as the middle dose with 3-fold lower and higher concentrations to establish a dose–response relationship. Piglets were housed in uniform environmental conditions to minimize confounders from cage location and measurement order. All piglets were housed in a controlled environment with a room temperature of 23–28 °C, relative humidity of 50–65%, and a 12 h light/12 h dark cycle. Ventilation and routine husbandry practices were consistent across all groups to minimize confounding effects. Following a 3-day acclimation period, the formal experiment was conducted for 28 days. The experimental diet was formulated for nursery piglets aged 35–80 days, free of antibiotics and any nationally prohibited substances. A basal antibiotic-free diet was used for all groups, and its ingredients and nutrient levels are presented in Table 1. At the end of the study, all piglets underwent only noninvasive sampling (anterior vena cava anticoagulant blood collection, anal swabs) with no sacrifice or euthanasia; all procedures were designed to minimize piglet discomfort and strictly comply with institutional animal welfare ethics.

Blood samples and feces were collected from all piglets at the start of the experiment (0 days), mid-experiment (14 days), and end of the experiment (28 days). Each piglet was weighed to determine body weight gain and feed conversion ratio (FCR). Body weight was individually weighed on day 0, 14, and 28 of the trial. Average daily gain (ADG) was calculated as the ratio of total weight gain to the number of days in each period. Daily feed intake was recorded precisely to calculate average daily feed intake (ADFI). FCR was defined as the ratio of ADFI to ADG.

All blood samples were used to determine serum immunoglobulin G (IgG) and cytokines. Antibody titers were detected using kits (CSFV for IDEXX, ME, USA; PRV for Beijing Jinnuobaitai Biotechnology Co., Ltd, Beijing, China). A piglet was defined as seropositive if its serum sample met the following criteria: CSFV antibody blocking rate ≥ 0.4 and PRV antibody S/N value ≤ 0.60. Cytokines including IL-1β, IL-2, IFN-γ, and TNF-α (Suzhou Sizhengbai Biotechnology Co., Ltd., Suzhou, China), IL-4 and IL-10 (Wuhan Huamei Biotechnology Co., Ltd, Wuhan, China.), and IFN-α (Jiangsu Jingmei Biotechnology Co., Ltd, Yancheng, China.) were analyzed with corresponding enzyme-linked immunosorbent assay (ELISA) kits with a Tecan Infinite F50 microplate reader. Fecal samples for intestinal flora analysis were subjected to 16S rRNA V3-V4 region high-throughput sequencing, which was outsourced to Shanghai Sangon Biotech Co., Ltd(Shangsha, China). The PCR amplification was performed with primers 338F (5′-ACTCCTACGGGA GGCAGCAG-3′) and 806R (5′-GGACTACHVGGGTWTCTAAT-3′), sequencing was conducted on the Illumina MiSeq PE300 platform, and all sequencing data were analyzed on the official cloud analysis platform of Sangon Biotech (https://ngs.sangon.com (accessed on 11 December 2023, 20 December 2023, and 03 January 2024.))

### 2.6. Statistical Analysis

Group allocation and experimental procedures were performed by dedicated staff aware of group assignments. Outcome evaluation and data analysis were conducted by investigators blinded to group allocation. All experimental data were statistically analyzed using SPSS 27.0 version software (IBM Corp., Armonk, NY, USA), and results are presented as mean ± standard deviation. The appropriate statistical tests were selected based on the experimental design and repeated measures as follows. For comparisons of normally distributed data across multiple groups, one-way analysis of variance (one-way ANOVA) was performed, followed by Tukey’s post-hoc test for pairwise comparisons between groups. For 4 levels of *G. alkanivorans* supplementation, polynomial regression analysis was additionally conducted to test the linear, quadratic, and cubic effects, so as to clarify the dose response relationship. For comparisons of non-normally distributed data or categorical data, Kruskal Wallis H test was used for multiple-group comparisons, and Mann Whitney U test was used for pairwise comparisons. For comparisons of repeated measures data, two-way repeated measures ANOVA was applied, with group as the between-subject factor and time as the within-subject factor. Post-hoc analysis for between-group and within-group effects were conducted using Bonferroni correction. All statistical analyses were performed using IBM SPSS Statistics 26.0.

The significance level for all statistical tests was set at α = 0.05, where *p* < 0.05 was defined as a statistically significant difference and *p* < 0.01 as a highly statistically significant difference. Exact *p*-values are reported for all between-group comparison results, and 95% confidence intervals are supplemented for key effect sizes, such as mean differences in indicators and inhibition rates, to quantify the uncertainty of estimates. Data distribution was tested to match statistical assumptions; non-normal/categorical data were analyzed with nonparametric tests accordingly.

## 3. Results

### 3.1. Determination of the Safe Concentration of G. alkanivorans

**Figure 1:**
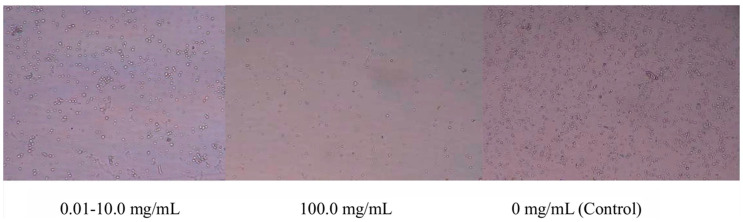
Safe drug concentration of *G. alkanivorans.* AMs were treated with *G. alkanivorans* at concentrations of 0.01–100.0 mg/mL, 0 mg/mL (control) for 24 h. Cytopathic effects and cell morphology were observed under a biological microscope at 1:100 magnification. n = 3 wells per group.

No obvious morphological alterations were observed in PAMs treated with *G. alkanivorans* at concentrations ranging from 0.01 to 10 mg/mL compared with the control group. Whereas obvious morphological changes were detected in PAMs exposed to 100 mg/mL *G. alkanivorans*. Thus, the maximal noncytotoxic concentration of the strain for PAMs was defined as 10 mg/mL (Figure 1).

Half Cytotoxic Concentration After treating PAMs with *G. alkanivorans* at gradient concentrations (100, 10, 1, 0.1, 0.01, 0.001 mg/mL) for 24 h, CCK-8 assay results revealed that cell viability remained unchanged significantly at concentrations ≤10 mg/mL, but decreased significantly at 100 mg/mL (Table 2).

**Figure 2:**
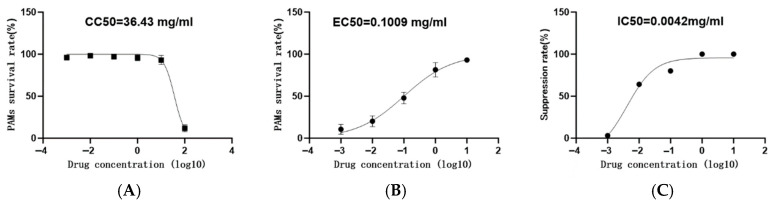
Dosage-survival fitting curve of *G. alkanivorans.* (A) CC_50_ curve of *G. alkanivorans* in PAMs. (**B**) EC_50_ curve of *G. alkanivorans* in PAMs. (**C**) IC_50_ curve of *G. alkanivorans* against PRRSV. Data are presented as mean ± SD, n = 3 wells per group.

ANOVA followed by Tukey’s post-hoc test confirmed significant differences between groups (*p* < 0.01). Additionally, polynomial regression analysis revealed significant linear (*p* < 0.01) and quadratic (*p* < 0.05) effects of *G. alkanivorans* dosage on PAMs viability, indicating a clear dose–response relationship. The CC_50_ value of *G. alkanivorans* for PAMs was subsequently calculated as 36.43 mg/mL using GraphPad Prism 6 software (Figure 2A).

**Half effective concentration.** After pretreatment of PAMs with gradient concentrations of *G. alkanivorans* (10, 1, 0.1, 0.01, 0.001 mg/mL), results showed that cell viability increased with increasing concentrations of the strain, reaching a maximum of 93% in the 10 mg/mL group (Table 3). ANOVA followed by Tukey’s post-hoc test confirmed significant differences between groups.

**Table 3:** Viability of PAMs in the EC_50_ assay determined by CCK-8 assay.

Dosage	0 (Ac)	0.001	0.01	0.1	1.0	10	*p*	Trend *p* (L/Q/C)
As	0.3400	0.1911	0.2423	0.2480	0.2670	0.3020	<0.01	<0.01/<0.01/<0.421
Ab	0.2330	0.1800	0.2210	0.1968	0.1800	0.2018	<0.01	<0.01/<0.01/<0.387
Viability %		10 ^a^	20 ^b^	48 ^c^	81 ^d^	93 ^e^	<0.01	<0.01/<0.01/<0.295

The viability rate of PAMs was calculated using the following formula: V% = (A_s_ − A_b_)/(A_c_ − A_b_), where A_s_, A_b_, and A_c_ represent the absorbance of the sample well, blank well, and control well, respectively. According to the instructions of the CCK-8 kit, a separate blank absorbance value (A_b_) was assigned to each drug group due to the intrinsic color of the test drugs. Data are presented as mean values (n = 3 wells per group). Different superscripts (a, b, c, d, e) within a row indicate significant differences among dosage groups (one-way ANOVA, *p* < 0.05, followed by Tukey’s post-hoc test). L/Q/C = Linear/Quadratic/Cubic. Polynomial regression analysis was performed to test for linear, quadratic, and cubic dose response effects of the treatments on PAM viability.

Polynomial regression analysis revealed significant linear (*p* < 0.01) and quadratic (*p* < 0.05) effects of *G. alkanivorans* dosage on PAMs viability, indicating a clear dose response relationship. Accordingly, the EC_50_ value of *G. alkanivorans* for PAMs was determined to be 0.1009 mg/mL using GraphPad Prism 6 software (Figure 2B).

**Half-Maximal Inhibitory Concentration** PRRSV copy numbers in infected PAMs were quantified by qPCR, with the results presented in Figure 2C. These data indicated that *G. alkanivorans* at concentrations of 0.001–1.0 mg/mL inhibited PRRSV replication to varying degrees, while concentrations of 1.0–10 mg/mL resulted in complete (100%) inhibition of viral replication. The half-maximal inhibitory concentration (IC_50_) of *G. alkanivorans* for PRRSV was subsequently determined to be 0.0043 mg/mL using GraphPad Prism 6 software.

### 3.2. Determination of Safety Concentration of G. alkanivorans Cell Wall Extract

**Figure 3:**
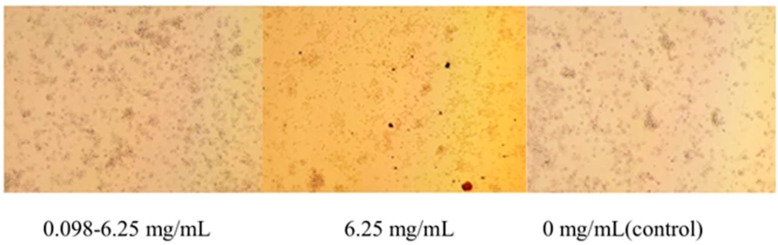
Safe concentration of *G. alkanivorans* cell wall extract. PAMs were treated with *G. alkanivorans* cell wall extract at concentrations of 0.098–6.25 mg/mL, 0 mg/mL (control) for 24 h. Cell morphology was observed under a biological microscope at 1:100 magnification. n = 3 wells per group. PAMs were treated with *G. alkanivorans* cell wall extract at concentrations of 0.098–6.25 mg/mL, 6.25 mg/mL, or 0 mg/mL (control) for 24 h. Cell morphology was observed under a biological microscope at 1:100 magnification. n = 6 wells per group. Scale bar = 100 µm.

Cytopathic effects were observed under a microscope and are presented below. At a concentration of 0.098–6.25 mg/mL, the *G. alkanivorans* cell wall extract did not induce obvious morphological changes in PAMs compared with the control group. In contrast, exposure to the *G. alkanivorans* cell wall extract at 25 mg/mL resulted in a pronounced cytopathic effect. Based on these findings, the maximal non-cytotoxic concentration of the *G. alkanivorans* cell wall extract was determined to be 6.25 mg/mL (Figure 3).

### 3.3. G. alkanivorans Cell Wall Extract Enhances Phagocytic Efficiency of PAMs Against PRRSV

**Figure 4:**
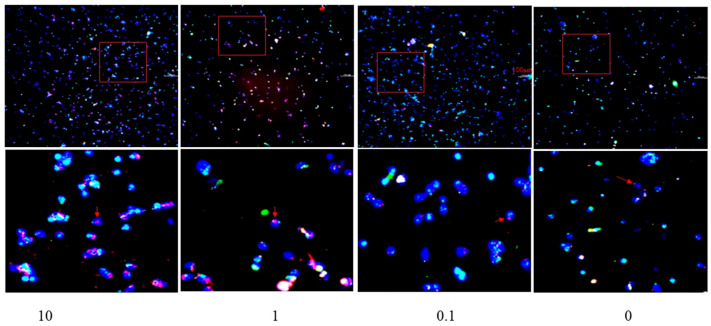
Photos of the fluorescence tracer test. PAMs were first co-incubated with *G. alkanivorans* cell wall extract at concentrations of 10 µg/mL (**left**), 1 µg/mL (**left-middle**), 0.1 µg/mL (**right-middle**), and 0 µg/mL (**right**) (control group), respectively. Subsequently, the PAMs were incubated with DID-labeled PRRSV (red, indicated by red arrows).The red boxes in the upper panoramic images indicate the corresponding regions shown at 4× magnification in the lower panels. For fluorescent staining, PAM nuclei were stained with DAPI (blue fluorescence), while PAM cell membranes were stained with β-antibody (green fluorescence). n = 3 wells per group.

**Figure 5:**
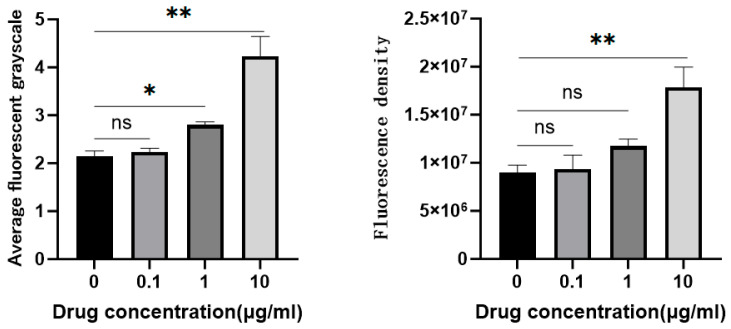
Fluorescence tracing results. PAMs were first co-incubated with *G. alkanivorans* cell wall extract at concentrations of 0 µg/mL, (control group), 0.1 µg/mL, 1 µg/mL, and 10 µg/mL, respectively. Subsequently, the PAMs were incubated with DID-labeled PRRSV. For fluorescent staining, PAM nuclei were stained with DAPI, while PAM cell membranes were stained with β-antibody. Fluorescence intensity was observed and photographed under a fluorescence microscope and quantified using ImageJ 1.8 version. Left: Mean fluorescence intensity. Right: fluorescence density map. ** *p* < 0.01, * *p* < 0.05, ns: *p* > 0.05. n = 3 wells per group.

Fluorescence tracing assays revealed that a higher concentration of *G. alkanivorans* cell wall extract was associated with increased PRRSV engulfment by PAMs (Figure 4). Specifically, with increasing concentration of *G. alkanivorans* cell wall extract (0, 0.1, 1.0, and 10 µg/mL), both the fluorescence density (0.89, 0.93, 1.17, and 1.78) and average fluorescence grayscale (2.14, 2.23, 2.80, and 4.24) showed a corresponding upward trend (Figure 5). Notably, at 10 µg/mL, the fluorescence intensity of the treatment group was 2.01-fold higher than that of the control group. These findings confirm that *G. alkanivorans* cell wall extract improves the phagocytic capacity of PAMs against PRRSV.

**Figure 6:**
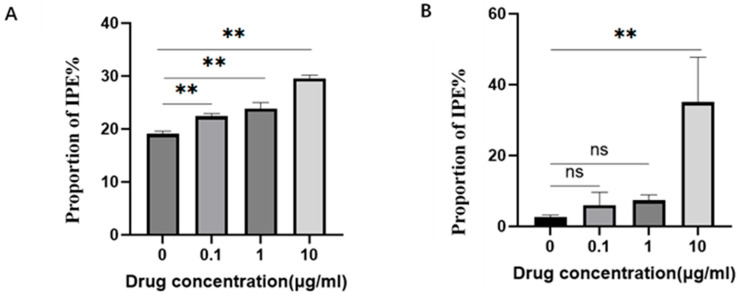
PAMs phagocytosis rate. After co-incubating PAMs with PRRSV, it was removed for all unengulfed free PRRSV particles adhering to the cell surface. Subsequently, PAMs were lysed, and their nucleic acids were extracted; the number of viral copies was then quantified using a qPCR kit. Finally, the viral phagocytosis rate of PAMs was calculated via the ΔΔCt method. (**A**). Proportion of increased phagocytic efficiency of PRRSV by PAM. (**B**). Proportion of increased phagocytic efficiency of *E. coli* by PAM; ** *p* < 0.01, ns: *p* > 0.05. n = 3 wells per group.

The number of phagocytosed PRRSV copies was quantified via qPCR, and the corresponding phagocytosis rate was calculated accordingly. With increasing concentrations of *G. alkanivorans* cell wall extract (0, 0.1, 1.0, 10 µg/mL), the PRRSV Ct values were 38.57, 38.34, 38.25, and 37.94, respectively. Compared with the control group (0 µg/mL), phagocytic efficiency of PAMs against PRRSV was increased by 19.08%, 22.38%, 23.82%, and 29.52% at the respective concentrations. These findings indicate that the *G. alkanivorans* cell wall extract enhances the phagocytic efficiency of PAMs against PRRSV in a dose dependent manner, which is consistent with the results of the fluorescence tracing assay (Figure 6A).

### 3.4. G. alkanivorans Cell Wall Extract Enhances Phagocytic Efficiency of PAMs Against E. coli

With increasing concentrations of *G. alkanivorans* cell wall extract (0, 0.1, 1.0, 10 µg/mL), the phagocytosed *E. coli* loads was 2.6 × 10^3^, 6.0 × 10^3^, 7.3 × 10^3^, and 3.5 × 10^4^ CFU/mL, respectively. Relative phagocytic efficiency at these concentrations was 2.31-, 2.81-, and 13.46-fold that of the control group (2.6 × 10^3^), respectively. Notably, a highly significant difference was observed between the high-dose group (10 µg/mL) and the control group (Figure 6B).

### 3.5. Average Daily Gain (ADG)

Regarding ADG of piglets, phase-specific differences were observed among groups supplemented with different doses of *G. alkanivorans* and the control group, as determined confirmed by one-way ANOVA followed by Tukey’s Post-Hoc Test.

In the 0–14 d phase, the control group had the highest ADG (0.824 kg/d), whereas the medium-(0.601 kg/d), low-(0.594 kg/d) and high-dose (0.577 kg/d) groups were significantly lower (*p* < 0.05), with no significant differences among the three treatment groups.

In the 14–28 d phase, *G. alkanivorans*-supplemented groups showed distinct advantages: the medium-dose group had the highest ADG (0.779 kg/d), followed by the low- (0.761 kg/d) and high-dose (0.743 kg/d) groups, whereas the control group had the lowest ADG (0.489 kg/d), with significant differences among all groups (*p* < 0.05).

For the entire 0–28 d trial period, the medium-dose group still displayed the highest ADG (0.690 kg/d), representing a 5.18% increase compared with the control group (0.656 kg/d, *p* < 0.05). The low-dose group (0.678 kg/d) showed a 3.35% increase (*p* > 0.05), while the high-dose group (0.655 kg/d) exhibited a 0.15% decrease compared with the control group, with no significant difference (*p* > 0.05). Polynomial regression analysis revealed significant quadratic effects (*p* < 0.05) of *G. alkanivorans* dosage on ADG in the 14–28 day phase, confirming a dose-dependent growth response.

In summary, *G. alkanivorans* exerted a phase dependent effect on piglet ADG: no growth-promoting effect was observed in the early stage (0–14 d), which may be attributed to microbial adaptation, whereas it significantly improved growth performance in the late stage (14–28 d), with medium-dose supplementation producing the most pronounced growth-promoting effect (Table 4).

### 3.6. Feed Conversion Ratio (FCR)

The effect of *G. alkanivorans* on FCR exhibited significant dose dependence and stage specificity throughout the piglet growth cycle, with detailed results as follows (Table 4). 0–14 days stage: The control group had the lowest FCR (1.475 ± 0.073). The medium-dose group showed a significantly increased FCR (2.076 ± 0.096), and the high-dose group also had a significantly higher FCR than the control group (2.107 ± 0.149). Additionally, the low-dose group had a significantly higher FCR (1.901 ± 0.104) than the control group (One-way ANOVA + Tukey’s test, all *p* < 0.05).

The 14–28 days stage: the control group had the highest FCR (2.566 ± 0.207). Both the low-dose group (1.677 ± 0.167) and the medium-dose group (1.650 ± 0.139) had significantly lower FCR than the control group, and the high-dose group (1.755 ± 0.132) also showed a significantly lower FCR than the control group (one-way ANOVA + Tukey’s test, all *p* < 0.05).

The 0–28 days full cycle: the control group had an FCR of 1.876 ± 0.036. The medium-dose group showed no significant difference in FCR (1.836 ± 0.076) compared to the control group, while the low-dose group had a significantly lower FCR (1.776 ± 0.085) than the control group, and the high-dose group had a significantly higher FCR (2.051 ± 0.145) than the control group (one-way ANOVA + Tukey’s test, *p* < 0.05).

In summary, *G. alkanivorans* exerted a dual effect on FCR, characterized by increased FCR in the early stage and decrease FCR in the later stage. The medium dose did not significantly affect FCR over the entire cycle, whereas the high dose resulted in a significant increase in FCR during the full cycle, suggesting that excessive supplementation may negatively affect long-term feed conversion efficiency in piglets.

### 3.7. Analysis of Antibody Positive Rates

During the 0–14 d phase, the positive rates of CSFV antibodies increased by 18.75% (low-dose group), 31.25% (medium-dose group), and 50.00% (high-dose group) in all experimental groups, respectively, whereas the control group showed a 25.00% decrease. These results indicate that the high-dose group of this bacterium exhibits a stronger capacity to activate the early humoral immune system. During 14–28 d phase, only the low-dose group maintained a continuous upward trend in CSFV antibody positive rates (37.50%). Over the entire period of days 0–28, the CSFV antibody positive rates increased in all experimental groups. Regarding PRV antibodies, the decrease in positive rates in all experimental groups was less pronounced than that in the control group during the 0–28 d period. This suggests that *G. alkanivorans* can attenuate the declining trend of PRV antibodies. During the 0–28 d period, serum cytokines levels were affected by *G. alkanivorans* and detailed analysis is described separately as follows (Table 5).

### 3.8. Analysis of Serum Cytokine Levels

Interleukin (IL)-1β: The medium-dose group (6.7 ± 13.07 pg/mL) was significantly higher than the control group (−48.06 ± 38.04 pg/mL) (*p* < 0.05), while the low-dose group (−117.51 ± 75.69 pg/mL) and high-dose group (−25.94 ± 25.55 pg/mL) showed no significant differences from the control group (Table 6).

IL-2: The low-dose group (−625.92 ± 161.09 pg/mL), medium-dose group (−57.35 ± 144.97 pg/mL), and high-dose group (−357.76 ± 177.62 pg/mL) were all significantly lower than the control group (4.43 ± 274.26 pg/mL) (all *p* < 0.01). The IL-2 content in all experimental groups was reduced by 14–192 times compared with the control group, suggesting that *G. alkanivorans* can induce functional remodeling of the immune response. In this study, while IL-2 levels decreased, the pro-inflammatory factor tumor necrosis factor (TNF)-α increased significantly, and the anti-inflammatory factor IL-10 decreased significantly in the high-dose group. These results suggest that the regulation of IL-2 by *G. alkanivorans* may be achieved by remodeling the balance of T cell subsets. Specifically, it may inhibit Th2 cell differentiation (IL-2 is a key factor for Th2 cell proliferation while promoting Th1 cell polarization (indirectly regulated by upregulating factors such as IFN-γ), ultimately leading to a staged-specific decrease in IL-2 levels. This change is not simply an inhibition of inflammation but a functional remodeling of the immune response [30].

TNF-α: the low-dose group (−13.85 ± 17.87 pg/mL), medium-dose group (14.77 ± 2.75 pg/mL), and high-dose group (18.11 ± 4.08 pg/mL) were all significantly higher than the control group (10.23 ± 3.23 pg/mL) (all *p* < 0.01). During the 0–28 d period, the TNF-α content in all experimental groups increased by 39–191% compared with the control group, indicating that *G. alkanivorans* can enhance the body’s antiviral capacity.

IL-10: the high-dose group (3.39 ± 2.92 pg/mL) was significantly lower than the control group (6.63 ± 2.17 pg/mL) (*p* < 0.05), while the low-dose group (2.49 ± 2.43 pg/mL) and medium-dose group (7.34 ± 1.95 pg/mL) showed no significant differences from the control group.

No significant differences were observed in the levels of other cytokines (IFN-α, IFN-γ, IL-4) among all groups (*p* > 0.05). In contrast, the concentrations of IL-1β, TNF-α, IFN-γ, IL-4, and IL-10 in the low-, medium- and high-dose groups showed distinct dynamic changes compared with the control group. Thus, although *G. alkanivorans* induced changes in cytokine profiles, the precise mechanism by which it regulates the functional activity of these cytokines remains to be further elucidated.

### 3.9. Alpha Diversity Analysis in Intestinal Gut Microbiota 

In 0–14 d phase, the out count, Shannon index and Chao index (indicators of intestinal microbiota alpha diversity) were all increased to varying degrees in each experimental group compared with the control group. In the 14–28 d phase, these indices were decreased in all experimental groups relative to the control group. However, over the entire 0–28 d trial period, the indices were again elevated to varying degrees in all experimental groups compared with the control group: Operational Taxonomic Unit (OUT) counts were increased by 11.94% (low dose), 9.10% (medium dose), and 10.53% (high dose); Shannon indices were increased by 29.43% (low dose), 8.25% (medium dose), and 5.96% (high dose); and Chao indices were increased by 14.63% (low dose), 9.46% (medium dose), and 10.01% (high dose) (Table 7).

### 3.10. Changes in Composition of Species of Intestinal Bacterial Phyla and Genera 

Compared with the control group, the relative abundance of Firmicutes exhibited a trend of first decreasing (0–14 d) and then increasing (14–28 d); however, it showed a downward trend over the entire 0-28 d trial period. Specifically, it decreased by 4.62-fold (Low), 8.6-fold (medium), and 17.83-fold (high), respectively. The relative abundance of bacteroides showed the opposite trend. Compared with the control group, it first increased (0–14 d) and then decreased (14–28 d), with a downward trend overall (0–28 d). Specifically, it decreased by 1.70-, 2.45-, and 3.07-fold in the low-, medium-, and high-dose groups, respectively (Table 8).

The relative abundances of Proteobacteria, Actinobacteria, and Bacteroidetes showed irregular fluctuations, which may be associated with the inhibitory effect of *G. alkanivorans* on pathogenic bacteria [31]. An increase in Bacteroidetes abundance may promote dietary fiber degradation [32].

Over the entire 0–28 d trial period, the relative abundance of Firmicutes in the high-dose group was −7.07, significantly lower than that in the control group (0.42, *p* < 0.05). The relative abundance of Bacteroidetes in the medium-dose group (6.17) was significantly higher than that in the control group (1.79, *p* < 0.05), and that in the high-dose group (7.29) was extremely significantly higher than that in the control group (*p* < 0.01). These results indicate that long-term supplementation of *G. alkanivorans* can significantly alter the relative abundances of Firmicutes and Bacteroidetes, with the high-dose group exerting the most prominent promoting effect on Bacteroidetes abundance.

In conclusion, the regulation of *G. alkanivorans* on the relative abundances of major intestinal bacterial phyla in piglets is obviously phase-dependent, with differential effects on different bacterial phyla at different growth stages; the medium- and high-dose groups exhibited more significant regulatory effects.

### 3.11. Firmicutes/Bacteroidetes (F/B) Ratio

Among all groups, the F/B ratio in the middle-dose group decreased the most significantly, followed by the high-dose group, whereas there was no significant difference between the low- dose group and the control group. Compared with the control group, the F/B ratios in all experimental group showed a trend of first decreasing (0–14 d) and then increasing (14–28 d). Overall, the entire 0–28 d trial period, the overall trends differed among groups: a decreasing trend was observed in the medium-dose group, while an increasing trend was noted in the low- and high-dose groups. Specifically, the F/B ratio decreased by 16.76-fold in the medium-dose group and increased by 15.19-fold and 16.76-fold in the low- and high-dose groups, respectively (Table 9).

## 4. Discussion

To evaluate the safety and antiviral efficacy of *G. alkanivorans*, we determined its CC_50_, EC_50_, and IC_50_ against PRRSV. The IC_50_ of *G. alkanivorans* (0.0043 mg/mL) was comparable to that of *Bacillus subtilis* (0.0038 mg/mL) and *Actinomycetes* (0.0009 mg/mL), confirming its strong capacity to inhibit PRRSV replication. Notably, *G. alkanivorans* completely suppressed PRRSV replication, further supporting its potent antiviral effect. The CC_50_ of *G. alkanivorans* reached as high as 36.43 mg/mL, which was significantly higher than those of *Lactobacillus* (3.776 mg/mL), *Bacillus subtilis* (0.4366 mg/mL), and *Actinomycetes* (0.2278 mg/mL) [33,34,35]. This indicates *G. alkanivorans* carries minimal cytotoxic risk of to host PAMs. Both the therapeutic index (CC_50_/EC_50_) and the viral inhibition index (CC_50_/IC_50_) were substantially higher than those of the aforementioned probiotics/antimicrobial agents, collectively confirming that *G. alkanivorans* exhibits high safety and strong antiviral activity, making it a promising candidate for PRRSV control.

To identify the key functional component of *G. alkanivorans*, we further investigated the effect of its cell wall extract on PAMs. First, the safe concentration of the *G. alkanivorans* cell wall extract was determined to be 6.25 µg/mL, as no significant PAM morphological changes were observed at this concentration, whereas marked cytopathic effects occurred at 25 µg/mL, consistent with previous reports on bacterial cell wall extracts [36]. Subsequent experiments demonstrated that the *G. alkanivorans* cell wall extract enhanced PAM phagocytic efficiency in a dose-dependent manner. Fluorescence tracing assays showed that with increasing extract concentration, both fluorescence density and average fluorescence grayscale of PRRSV-engulfed PAMs increased, with the 10 µg/mL group exhibiting 2.01-fold higher fluorescence intensity than the control. QPCR quantification further confirmed that the phagocytic efficiency of PAMs against PRRSV increased with compared to the control, with the highest efficiency observed at 10 µg/mL. CFU counting revealed that the phagocytic efficiency of the high-dose, medium-dose, and low-dose extract groups was 13.13-2.25-fold higher than that of the control, respectively, with a highly significant difference in the high-dose group. These results align with previous studies showing that bacterial components, such as muramyl dipeptide, can induce macrophage autophagy to promote pathogen clearance [37] and that bacteria directly activate macrophage metabolic activity to enhance phagocytic function [38]. TNF-α can positively regulate the phagocytic ability of porcine alveolar macrophages (PAMs) by promoting PAM polarization toward the M1 phenotype, upregulating phagocytosis-related molecules, and enhancing pathogen recognition and clearance. Collectively, our data confirm that the *G. alkanivorans* cell wall is a key functional component mediating the enhancement of PAM phagocytosis.

One of the primary objectives of this experiment was to evaluate the efficacy of *G. alkanivorans* in modulating PAM-mediated phagocytosis of PRRSV. The results demonstrated that *G. alkanivorans* enhances the efficiency of PRRSV phagocytosis by PAMs. Overall, this outcome is more beneficial than harmful, as it facilitates viral clearance and mitigates PRRSV infection. This conclusion is supported by the infection characteristics of PRRSV, as outlined below: (1) PRRSV primarily enters macrophages through receptor-mediated endocytosis, which constitutes the initial step in its infection and replication cycle. (2) A core function of macrophages is to phagocytose degrade, and clear pathogens. (3) The promotion of viral phagocytosis by bioactive agents (e.g., *G. alkanivorans*) accelerates the clearance of free extracellular PRRSV, thereby reducing the likelihood of the virus infecting new cells. (4) This effect is equivalent to enhancing the body’s innate immune defense capacity, which helps control viremia and alleviate clinical symptoms. Although the virus attempts to replicate after entering macrophages, the overall effect of promoting phagocytosis remains more beneficial than harmful, as it accelerates the process of viral clearance and limits infection progression

To evaluate the in vivo efficacy of *G. alkanivorans*, we monitored piglet weight gain and FCR over 28 days, which revealed a stage-dependent pattern. In the pre-experiment (Days 0–14), weight gain in all *G. alkanivorans*-supplemented groups were significantly lower (*p* < 0.01) than that in the control group, with a 27–30% reduction. FCR in the experimental groups was also inferior to the control (28.7–42.7% lower efficiency). This transient growth inhibition is consistent with findings from Mycobacterium phlei supplementation [39], likely due to a temporary stress response in piglets during the initial colonization of exogenous bacteria, characterized by transient intestinal mucosal barrier activation, mild pro-inflammatory cytokine (IL-6, IL-1β) elevation, and reduced nutrient digestibility as the host immune system recognizes and adapts to the exogenous microbial strain [32]. In the later experiment (Days 14–28), the growth performance of the *G. alkanivorans* groups significantly exceeded that of the control (*p* < 0.01): weight gain increased by 49.2–59.6% and FCR in the experimental groups (≥0.5699) was significantly higher than the control (*p* < 0.05). This improvement may be attributed to the establishment of intestinal homeostasis after *G. alkanivorans* colonization, which modulates the intestinal flora structure to increase beneficial bacteria abundance, enhances intestinal tight junction protein expression to improve nutrient absorption, and regulates anti-inflammatory cytokine secretion to alleviate chronic low-grade inflammation, thereby enhances nutrient utilization efficiency [40]. In the full experiment (Days 0–28), the medium-dose group exhibited the highest weight gain, which was significantly higher than the control (*p* < 0.05). Notably, the high-dose group had the highest FCR (2.051 ± 0.145), suggesting that excessive *G. alkanivorans* supplementation may interfere with nutritional metabolism, which was consistent with the document [41]. These results indicate that *G. alkanivorans* supplementation can improve piglet growth performance, with an optimal dose of 2.0 kg/ton.

We further analyzed the effect of *G. alkanivorans* on the antibody positive rates of CSFV and PRV to evaluate its impact on humoral immunity. Over 0–14 d, the CSFV antibody positive rate increased by 18.75–50.00% in the *G. alkanivorans* groups, whereas it decreased by 25.00% in the control, indicating that high-dose *G. alkanivorans* strongly activates the early humoral immune response. Over 14–28 d, only the low-dose group maintained a continuous increase (37.50%). Over the entire 28-day trial period, all *G. alkanivorans* groups showed increased CSFV antibody positive rates, which is consistent with the immune-enhancing effects of probiotics reported by the document [42]. Over days 0-28, the PRV antibody positive rate decreased by only 12.50% (low dose), 18.75% (medium dose), and 18.75% (high dose) in the *G. alkanivorans* groups, compared to a 31.25% decrease in the control. This suggests that *G. alkanivorans* can delay the decline in PRV antibody levels, potentially enhancing the durability of vaccine-induced humoral immunity, aligning with the document [43]. The differential humoral responses to CSF and PR vaccines are primarily attributed to: (1) CSFV induces a fragile, short-lived antibody response due to weak neutralizing epitope presentation, limited B cell activation caused by N pro-mediated IFN-I suppression, and insufficient long-lived plasma cell formation resulting from virus-induced lymphocyte apoptosis, which makes it highly susceptible to *G. alkanivorans* extract-mediated adjuvant enhancement [44]. In contrast, PRV establishes latent infection in sensory neurons, where low-level viral reactivation continuously stimulates immune surveillance and sustains humoral immune memory via LAT-regulated latency maintenance and memory B cell re-stimulation, independently of external adjuvants, thus rendering it insensitive to the immunostimulatory effect of *G. alkanivorans* extract [45]. (2) The CSF vaccine’s simple antigen structure (facilitating extract-induced Dendritic Cell presentation) vs. the PR vaccine’s complex antigens and pre-existing adjuvants. (3) Dose-dependent modulation of B cell function, which only impacts CSF-specific responses. These findings highlight the importance of pathogen/vaccine-specificity when evaluating the efficacy of microbial immunostimulants in livestock [46,47].

Cytokine levels reflect the status of cellular immunity and inflammation responses. Our results showed that *G. alkanivorans* differentially regulates cytokine production in piglets over the 0–28 d trial period. In terms of antiviral and anti-inflammatory effects, IFN-α levels increased by 39–191% in all *G. alkanivorans* groups compared to the control, which is consistent with the antiviral cellular immunity enhancement observed in *Mycobacterium granis* supplementation [48], confirming that *G. alkanivorans* strengthens the body’s antiviral capacity. In contrast, IL-2 levels were reduced by 14-192-fold in the *G. alkanivorans* groups (with a significant difference in the low-dose group), suggesting that *G. alkanivorans* may inhibit excessive inflammatory responses. In regard to Th2 polarization and immune homeostasis, the increase in IL-4 levels (a Th2-type cytokine) in the *G. alkanivorans* groups, coupled with reduced IL-2 (a Th1-type cytokine), implies that *G. alkanivorans* may alleviate immune hyperactivity through Th2 polarization, which is consistent with the previous document [49]. Additionally, TNF-α and IL-1β levels were higher in the *G. alkanivorans* groups than that in the control, which aligns with the activation of the Toll-like receptor pathway by probiotics [50]. Regarding unresolved changes in cytokine function, IL-1β, TNF-α, IFN-γ, IL-4, and IL-10 exhibited distinct trends across the different *G. alkanivorans* dose groups. While these changes indicate that *G. alkanivorans* modulates the cytokine network, the specific mechanisms by which *G. alkanivorans* alters the functions of individual cytokines require further investigation.

The regulatory effects of *G. alkanivorans* on cytokines, such as IL-2 and IFN-α, may be mediated through a multi-pathway mechanism of the gut microbiota-immune axis, which can be explained by the following pathways. 1. Activation of the toll-like receptors (TLR) Signaling Pathway. As a probiotic, the cell wall components of *G. alkanivorans*, such as lipopolysaccharide analogs and peptidoglycan, can be recognized by TLRs on the surface of porcine intestinal epithelial cells and immune cells. This recognition activates the downstream Myeloid Differentiation Primary Response 88-Nuclear Factor-Kappa B (NF-κB) signaling cascade, thereby promoting the transcription and secretion of Th1-type cytokines such as IL-2. Meanwhile, TLR signaling activation can upregulate IFN-α expression, further enhancing the body’s innate antiviral immune response [51]. 2. Immunomodulation mediated by Gut microbiota metabolites. *G. alkanivorans* can modulate the gut microbiota structure through its metabolic activities, promoting the production of short-chain fatty acids. Short-chain fatty acids activate G protein-coupled receptors on immune cells, which subsequently inhibits histone deacetylase activity and reduces NF-κB phosphorylation, thereby suppressing excessive pro-inflammatory cytokine secretion while promoting the production of antiviral cytokines such as IFN-α [52]. 3. Regulation of Immune Cell Subset Polarization. *G. alkanivorans* can promote Th1 cell polarization (secreting IL-2, IFN-γ) and NK cell activation (secreting IFN-α) through interactions with intestinal mucosal immune cells. For example, its metabolites can induce dendritic cells to secrete IL-12, which in turn drives naive T cells to differentiate into the Th1 phenotype, enhancing cellular immune responses [53]. Meanwhile, NK cells are stimulated by IFN-α, and their cytotoxic effects are activated, further strengthening antiviral immunity [54]. In summary, *G. alkanivorans* may regulate cytokines such as IL-2 and IFN-α through the synergistic action of TLR signal activation-microbiota metabolite mediation-immune cell polarization pathways. This mechanism also provides a theoretical basis for its potential in immune enhancement and antiviral applications in pigs.

The expression levels of the four pro-inflammatory cytokines, including TNF-α, IFN-α, IFN-γ, and IL-1β, were significantly upregulated in the experimental groups. The upregulation may promote the polarization of PAMs towards the M1 phenotype. Specifically, TNF-α and IFN-γ can activate the NF-κB and Janus Kinase-Signal Transducer and Activator of Transcription signaling pathways in PAMs, respectively, upregulating the expression of M1 phenotype marker molecules in PAMs [55]. This further enhances the phagocytic capacity of PAMs against pathogens, such as PRRSV and *E. coli*, which is consistent with the characteristics of M1-type macrophages that exhibit enhanced phagocytic and bactericidal functions [56]. On this basis, the enhanced phagocytic capacity of PAMs can promote the improvement of their antigen-presenting ability: after PAMs phagocytose and clear pathogens (PRRSV, *E. coli*), they degrade pathogen antigens into small-molecule antigen peptides, which then combine to major histocompatibility complex class II molecules on the surface of PAMs and present them to naive T lymphocytes (CD4^+^ T cells). This process can effectively activate the proliferation and differentiation of T helper cells, further promote the activation and maturation of B lymphocytes, enhance the secretion of specific antibodies by B cells, and ultimately lead to the enhancement of PAM-mediated immune response and a significant increase in antibody levels in piglets [57].

Intestinal microbiota diversity and composition are critical for nutrient absorption and immune regulation. Our alpha diversity and taxonomic analysis yielded key insights. For alpha diversity, OTUs number, Chao index (richness), and Shannon index (diversity) were higher in all *G. alkanivorans*-treated groups than in the control during 0–14 d, indicating that strain enhances early intestinal microbiota richness and diversity. During 14–28 d, these indices decreased in all groups, but the rate of decline was slower in the *G. alkanivorans* groups. This microbiota-stabilizing effect aligns with the reported function of licorice polysaccharides (a prebiotic) in slowing intestinal microbiota diversity in weaned piglets [58]. Over the entire 28-day period, the *G. alkanivorans* groups still showed increased diversity: OTUs (+10.53–11.94%), Shannon index (+5.96–29.43%), and Chao index (+9.46–14.63%), confirming long-term benefits of *G. alkanivorans* on microbiota diversity. For phylum-level compositional changes, Firmicutes and Bacteroidetes are the dominant phyla involved in immune regulation and nutrient absorption. Compared to the control, the relative abundance of Firmicutes in the *G. alkanivorans* groups showed a trend of initial decrease (Days 0–14) followed by increase (Days 14–28), but an overall decrease (4.62–17.83-fold) over the 28-day period. The relative abundance of Bacteroidetes displayed the opposite transient pattern (initial increase, then decrease) but also an overall decrease (1.70-fold–3.07-fold). The increase in Bacteroidetes during the early stage may promote dietary fiber degradation, whereas irregular changes in Proteobacteria and Actinobacteria may be associated to pathogen inhibition [59]. The F/B ratio is a key indicator of intestinal health, with higher ratios associated with disrupted intestinal homeostasis [60]. It serves as an index reflecting the degree of intestinal microbiota dysbiosis, with a higher F/B ratio corresponding to a poorer intestinal health [60].

All *G. alkanivorans* groups showed a transient F/B ratio decrease (days 0–14) followed by an increase (days 14–28). Over the 28-day trial, the medium-dose group exhibited a significant 16.76-fold F/B ratio reduction, whereas the low- and high-dose groups showed 15.19- and 16.76-fold increases. Notably, the reduced F/B ratio in the medium-dose group indicates improved intestinal health, consistent with the beneficial effects of Astragalus polysaccharides and *Bacillus subtilis* on this ratio [61,62].

Several limitations of this study should be noted. First, the precise molecular targets and signaling cascades by which *G. alkanivorans* regulates PRRSV immunity remain unclear. Second, its optimal dosage, administration route, and long-term biosafety in commercial pig farming need validation. Third, interactions with other vaccines or feed additives are unexplored. Future research will focus on: (1) identifying the strain’s key functional components; (2) clarifying their molecular mechanisms in regulating host immunity; (3) conducting field trials for practical application; and (4) developing combination strategies with vaccines to enhance pig disease resistance.

## 5. Conclusions

This study confirms that *G. alkanivorans* is safe and exerts potent anti-PRRSV activity by enhancing the phagocytic function of PAMs, with its cell wall serving as a key functional component. In vivo, dietary supplementation with *G. alkanivorans* boosts humoral immunity, modulates serum cytokine profiles, optimizes intestinal microbiota, and improves growth performance in piglets. Notably, a medium dosage (2.0 kg/ton diet) of *G. alkanivorans* was found to be the most effective, as it significantly increased average daily gain (ADG) by 5.18% (*p* < 0.05) compared to the control, while also enhancing serum immunoglobulin levels and promoting the growth of beneficial gut microbiota. These findings provide a solid theoretical foundation for the application of *G. alkanivorans* as a novel antimicrobial and growth-promoting agent.

## Figures and Tables

**Table 1:** Composition and nutrient levels of the experimental diet (%).

Ingredients	Content	Ingredients	Content	Nutrient	Content
Corn	65.00	Limestone	1.00	Crude protein (≥%)	17.00
Extruded soybean meal	20.00	Dicalcium phosphate	1.20	Lysine (≥%)	1.20
Fish meal	2.00	NaCl	0.30	Methionine (≥%)	0.40
Whey powder	3.00	Antibiotic-free premix	3.50	Vitamin A (IU/kg)	12,000
Wheat bran	4.00	Total	100.00	Vitamin D_3_ (IU/kg)	2500
				Vitamin E (mg/kg)	30
				Digestible energy (≥MJ/kg)	13.50

**Table 2:** Viability of PAMs in the CC_50_ assay determined by CCK-8 assay.

Dosage	0 (Ac)	0.001	0.01	0.1	1.0	10	100	*p*	Trend *p* (L/Q/C)
As	0.7280	0.7330	0.7400	0.7438	0.8088	1.2062	2.9684	<0.01	<0.01/<0.01/<0.215
Ab	0.7080	0.7130	0.7210	0.7250	0.7900	1.1880	2.9660	<0.01	<0.01/<0.01/<0.198
Viability %		100 ^a^	95 ^a^	94 ^a^	94 ^a^	91 ^b^	12 ^c^	<0.01	<0.01/<0.01/<0.302

Note: The viability rate of PAMs was calculated as V% = (As − Ab)/(Ac − Ab), where As, Ab, and Acc are the absorbances of the sample, blank, and control wells, respectively. A separate Ab was assigned to each drug group due to the test drugs’ intrinsic color. Data are presented as mean values (n = 3 wells per group). Different superscripts (a, b, c) within a row indicate significant differences among dosage groups (one-way ANOVA, *p* < 0.05, followed by Tukey’s post-hoc test). L/Q/C = Linear/Quadratic/Cubic. Polynomial regression analysis was performed to test for linear, quadratic, and cubic dose response effects of the treatments on PAM viability.

**Table 4:** Statistical comparison of *G. alkanivorans* on weight gain and FCR in piglets.

Item	Group	Low	Medium	High	Control	*p*	Trend *p* (L/Q/C)
Weight (kg)	0	14.66 ± 0.75 ^a^	14.27 ± 0.60 ^a^	15.05 ± 1.00 ^a^	14.41 ± 0.60 ^a^	0.892	0.915/0.782/0.654
	14	22.97 ± 0.98 ^b^	22.69 ± 0.53 ^b^	23.13 ± 0.93 ^b^	25.94 ± 0.77 ^a^	<0.001	<0.001/0.621/0.489
	28	33.63 ± 1.25 ^a^	33.59 ± 1.23 ^a^	32.22 ± 1.85 ^a^	32.78 ± 0.79 ^a^	0.614	0.587/0.423/0.711
Weight gain (kg)	0–14	8.31 ± 0.45 ^b^	8.42 ± 0.39 ^b^	8.08 ± 0.57 ^b^	11.53 ± 0.57 ^a^	<0.001	<0.001/0.890/0.567
	14–28	10.66 ± 1.06 ^b^	10.91 ± 0.92 ^b^	10.40 ± 0.78 ^b^	6.84 ± 0.55 ^a^	<0.001	<0.001/0.734/0.698
	0–28	18.97 ± 0.91 ^a^	19.33 ± 0.8 ^a^	18.33 ± 0.43 ^a^	18.38 ± 0.35 ^a^	0.528	0.496/0.387/0.821
ADG (kg/d)	0–14	0.594 ± 0.032 ^b^	0.601 ± 0.028 ^b^	0.577 ± 0.041 ^b^	0.824 ± 0.041 ^a^	<0.001	<0.001/0.901/0.612
	14–28	0.761 ± 0.076 ^bA^	0.779 ± 0.066 ^bA^	0.743 ± 0.056 ^bA^	0.489 ± 0.039 ^a^	<0.001	<0.001/0.678/0.590
	0–28	0.678 ± 0.033 ^a^	0.690 ± 0.029 ^a^	0.655 ± 0.015 ^a^	0.656 ± 0.013 ^a^	0.417	0.398/0.276/0.754
FCR (%)	0–14	1.901 ± 0.104 ^bB^	2.076 ± 0.096 ^bB^	2.107 ± 0.149 ^bB^	1.475 ± 0.073 ^a^	<0.001	<0.001/0.812/0.645
	14–28	1.677 ± 0.167 ^bA^	1.650 ± 0.139 ^bA^	1.755 ± 0.132 ^bA^	2.566 ± 0.207 ^a^	<0.001	<0.001/0.765/0.589
	0–28	1.776 ± 0.085 ^aA^	1.836 ± 0.076 ^aA^	2.051 ± 0.145 ^aB^	1.876 ± 0.036 ^a^	<0.05	<0.05/0.032/0.478

Note: Data are presented as mean ± SD (n = 16 piglets per group). For the same row, different lowercase letters indicate significant differences among groups (*p* < 0.05). A, B: advantage or disadvantage vs. control. ADG 0–28 d: Low (+3.35%, *p* = 0.231), Medium (+5.18%, *p* = 0.032), High (−0.15%, *p* = 0.982). ADG 0–14 d: Low (−27.91%, *p* < 0.001), Medium (−27.06%, *p* < 0.001), High (−29.98%, *p* < 0.001). ADG 14–28 d: Low (+55.62%, *p* < 0.001), Medium (+59.30%, *p* < 0.001), High (+51.94%, *p* < 0.001). Polynomial regression was used to test linear, quadratic, and cubic dose effects.

**Table 5:** Statistical comparison of *G. alkanivorans* on antibody positive rate against CSFV and PRV.

Group	Against	Low (%)	Medium (%)	High (%)	Control (%)	*p*	Trend *p* (L/Q/C)
0–14	CSFV	18.75 *^ab^	31.25 ^ab^	50.00 ^a^	−25.00 ^b^	0.028	0.035/0.122/0.418
	PRV	18.75 ^a^	−43.75 ^b^	−6.25 ^ab^	−43.75 ^b^	0.012	0.018/0.089/0.365
14–28	CSFV	37.50 ^a^	−43.75 ^b^	−25.00 ^ab^	0.00 ^ab^	0.009	0.011/0.067/0.298
	PRV	−37.50 ^b^	25.00 ^a^	−6.25 ^ab^	12.50 ^b^	0.015	0.021/0.078/0.342
0–28	CSFV	56.25 ^a^	−12.50 ^b^	25.00 ^ab^	−25.00 ^b^	0.005	0.007/0.042/0.211
	PRV	−18.75 ^ab^	−18.75 ^ab^	−12.50 ^a^	−31.25 ^b^	0.033	0.041/0.105/0.389

* This value is calculated as the average antibody positive rate of the 16 pigs in the low-dose group on day 14 of the experiment minus their average antibody positive rate on day 0 of the experiment. Antibody positivity rate is the percentage of seropositive piglets in each group (n = 16 per group), determined by ELISA with kit-specified OD cutoffs. Data are presented as mean values. Different superscripts (a, b) within a row indicate significant differences among groups (one-way ANOVA, *p* < 0.05, followed by Tukey’s post-hoc test). L/Q/C = Linear/Quadratic/Cubic. Polynomial regression analysis was performed to test for linear, quadratic, and cubic dose response effects of *G. alkanivorans* on antibody positive rates against CSFV and PRV.

**Table 6:** Statistical comparison of *G. alkanivorans* on cytokine level in piglet serum (pg/mL) on 0–28 day.

Group	Low	Medium	High	Control	*p*	Trend *p* (L/Q/C)
IL-1β	−117.51 ± 75.69 ^b^	6.7 ± 13.07 ^a^	−25.94 ± 25.55 ^ab^	−48.06 ± 38.04 ^ab^	0.01	0.018/0.076/0.342
IL-2	−625.92 ± 161.09 ^b^	−57.35 ± 144.97 ^a^	−357.76 ± 177.62 ^ab^	4.43 ± 274.26 ^a^	<0.001	<0.001/<0.0018/0.289
TNF-a	−13.85 ± 17.87 ^b^	14.77 ± 2.75 ^a^	18.11 ± 4.08 ^a^	10.23 ± 3.23 ^a^	<0.001	<0.001/<0.0018/0.315
IFN-a	4.10 ± 6.21 ^a^	−2.74 ± 1.39 ^a^	2.63 ± 1.77 ^a^	−4.51 ± 1.08 ^a^	0.623	0.587/0.421/0.712
IFN-γ	−66.18 ± 69.36 ^b^	73.01 ± 59.64 ^a^	−211.84 ± 168.65 ^c^	−61.1 ± 231.72 ^b^	<0.001	<0.001/<0.0018/0.267
IL-4	−6.58 ± 4.39 ^a^	0.46 ± 2.44 ^a^	−4.12 ± 3.05 ^a^	−3.81 ± 3.53 ^a^	0.418	0.392/0.276/0.654
IL-10	2.49 ± 2.43 ^b^	7.34 ± 1.95 ^a^	3.39 ± 2.92 ^ab^	6.63 ± 2.17 ^a^	0.021	0.028/0.089/0.387

Data are presented as mean ± SD (*n* = 16 piglets per group). Different superscripts (a, b, c) within a row indicate significant differences among groups (one-way ANOVA, *p* < 0.05, followed by Tukey’s post-hoc test). L/Q/C = Linear/Quadratic/Cubic. Polynomial regression analysis was performed to test for linear, quadratic, and cubic doseresponse effects of *G. alkanivorans* on serum cytokine levels. It indicates a significant difference compared with the control group (*p* < 0.01).

**Table 7:** Statistical comparison of *G. alkanivorans* on intestinal gut microbiota.

Group	Item	Low	Medium	High	Control	*p*	Trend *p* (L/Q/C)
0–14	OTUs	2930 ± 187.53 ^b^	3190.5 ± 496.75 ^ab^	3238.75 ±3 63.96 ^a^	3004.75 ± 175.78 ^ab^	0.036	0.042/0.118/0.456
	Shannon	0.8232 ± 0.12 ^b^	0.9019 ± 0.45 ^ab^	1.0778 ± 0.13 ^a^	0.6326 ± 0.1144 ^b^	0.029	0.035/0.092/0.389
	Chao	2825.42 ± 172.95 ^ab^	3075.14 ± 490.2 ^a^	3109.38 ± 376.59 ^a^	2871 ± 187.5 ^ab^	0.041	0.048/0.105/0.423
14–28	OTUs	3744.75 ± 153.03 ^a^	3837.50 ± 386.6 ^a^	3866.00 ± 359.61 ^a^	3775.00 ± 207.59 ^a^	0.892	0.876/0.798/0.654
	Shannon	−0.6715 ± 0.0813 ^a^	−0.5930 ± 0.34 ^a^	−0.6219 ± 0.08 ^a^	−0.4502 ± 0.12 ^a^	0.574	0.543/0.489/0.721
	Chao	3692.67 ± 153.06 ^a^	3793.37 ± 387.2 ^a^	3824.06 ± 360.41 ^a^	3736.51 ± 213.92 ^a^	0.765	0.732/0.689/0.811
0–28	OTUs	−1488.5 ± 268.09 ^a^	−1536.5 ± 193.89 ^a^	−1512.25 ± 54.14 ^a^	−1690.25 ± 63.52 ^a^	0.218	0.196/0.178/0.698
	Shannon	−3.3068 ± 1.2203 ^a^	−4.2992 ± 0.27 ^a^	−4.3964 ± 0.16 ^a^	−4.6857 ± 0.16 ^a^	0.345	0.312/0.287/0.756
	Chao	−1628.2 ± 289.89 ^a^	−1726.9 ± 197.93 ^a^	−1716.24 ± 53.87 ^a^	−1907.24 ± 55.70 ^a^	0.189	0.167/0.152/0.789

Data are presented as mean ± SD (*n* = 16 piglets per group). Different superscripts (a, b) within a row indicate significant differences among groups (One-way ANOVA, *p* < 0.05, followed by Tukey’s post-hoc test). L/Q/C = Linear/Quadratic/Cubic. Polynomial regression analysis was performed to test for linear, quadratic, and cubic dose-response effects of *G. alkanivorans* on gut microbial diversity indices (OTUs, Shannon, Chao). OTU: It is a classification unit for microbial community analysis (not a quantitative unit with physical dimensions). Each OTU represents a group of microbial sequences with ≥97% similarity, reflecting the number of distinct microbial taxa in the intestinal microbiota. Shannon Index: A dimensionless index quantifying microbial diversity, integrating both species richness (number of taxa) and evenness (relative abundance of taxa). Higher values indicate greater diversity. Chao Index: Another dimensionless index estimating microbial species richness, particularly sensitive to rare taxa. Higher values reflect a more diverse community.

**Table 8:** Statistical comparison of *G. alkanivorans* on changes in intestinal flora (%).

Group	Item	Low	Medium	High	Control	*p*	Trend *p*-(L/Q/C)
0–14	Firmicutes	0.48 ± 4.59 ^ab^	−6.8 ± 3.14 ^b^	−4.74 ± 4.16 ^ab^	3.75 ± 3.51 ^a^	0.032	0.038/0.105/0.421
	Bacteroides	−1.76 ± 4.23 ^b^	7.65 ± 2.61 ^a^	3.26 ± 4.42 ^ab^	−3.98 ± 4.19 ^b^	0.018	0.023/0.089/0.367
	Actinomycetes	−1.03 ± 0.38 ^a^	−2.71 ± 1.59 ^a^	−1.11± 0.29 ^a^	−1.65 ± 0.51 ^a^	0.624	0.598/0.476/0.712
	Proteobacteria	1.31 ± 0.05 ^b^	1.24 ± 0.1 ^a^	1.03 ± 0.07 ^a^	0.77 ± 0.54 ^a^	<0.001	<0.001/<0.001/0.289
	Spiral Door	−0.19 ± 0.41 ^a^	−0.35 ± 0.37 ^a^	0.33 ± 0.24 ^a^	0.0 ± 0.09 ^a^	0.417	0.392/0.276/0.654
14–28	Firmicutes	−1.99 ± 0.36 ^ab^	3.61 ± 5.79 ^a^	−2.32 ± 3.45 ^ab^	−3.34 ± 3.53 ^b^	0.027	0.033/0.098/0.398
	Bacteroides	4.81 ± 0.66 ^a^	−1.48 ± 5.42 ^b^	4.02 ± 3.52 ^a^	5.77 ± 3.69 ^a^	0.015	0.021/0.076/0.342
	Actinomycetes	−0.6 ± 0.16 ^a^	−0.49 ± 0.32 ^a^	−0.26 ± 0.15 ^a^	−0.61 ± 0.4 ^a^	0.589	0.562/0.431/0.734
	Proteobacteria	−1.27 ± 0.22 ^a^	−1.43 ± 0.08 ^a^	−1.06 ± 0.13 ^a^	−1.46 ± 0.04 ^a^	0.612	0.587/0.465/0.721
	Spiral Door	0.12 ± 0.45 ^a^	0.26 ± 0.02 ^a^	0.25 ± 0.09 ^a^	0.48 ± 0.22 ^a^	0.485	0.461/0.328/0.689
0–28	Firmicutes	−1.52 ± 4.83 ^ab^	−3.19 ± 3.29 ^ab^	−7.07 ± 3.1 ^b^	0.42 ± 1.62 ^a^	0.023	0.029/0.082/0.315
	Bacteroides	3.05 ± 4.46 ^ab^	6.17 ± 4.54 ^a^	7.29 ± 3.36 ^a^	1.79 ± 1.85 ^b^	0.011	0.016/0.067/0.289
	Actinomycetes	−1.63 ± 0.26 ^a^	−3.21 ± 1.59 ^a^	−1.37 ± 0.23 ^a^	−2.26 ± 0.3 ^a^	0.576	0.551/0.423/0.718
	Proteobacteria	0.05 ± 0.24 ^a^	−0.19 ± 0.04 ^a^	−0.03 ± 0.15 ^a^	−0.69 ± 0.55 ^a^	0.634	0.609/0.487/0.742
	Spiral Door	−0.08 ± 0.33 ^a^	−0.09 ± 0.38 ^a^	0.58 ± 0.33 ^a^	0.47 ± 0.18 ^a^	0.429	0.405/0.291/0.667

Data are presented as mean ± SD (*n* = 16 piglets per group). Different superscripts (a, b) within a row indicate significant differences among groups (One-way ANOVA, *p* < 0.05, followed by Tukey’s post-hoc test). L/Q/C = Linear/Quadratic/Cubic. Polynomial regression analysis was performed to test for linear, quadratic, and cubic dose-response effects of *G. alkanivorans* on the relative abundance of gut microbiota phyla. It indicates a significant difference compared with the control group (*p* < 0.05).

**Table 9:** Statistical results of changes in *F/B* values.

Group	Low	Medium	High	Control	*p*	Trend *p* (L/Q/C)
0–14	1.139 ± 1.918 ^aA^	−6.143 ± 5.028 ^aA^	−1.268 ± 1.677 ^a A^	1.373 ± 1.565 ^a^	0.124	0.142/0.218/0.567
14–28	−1.447 ± 0.676 ^aB^	−0.288 ± 1.087 ^aB^	−0.833 ± 0.795 ^aB^	−1.735 ± 1.443 ^a^	0.389	0.365/0.298/0.689
0–28	−0.307 ± 1.2946 ^aA^	−6.431 ± 5.995 ^bA^	−2.101 ± 1.3616 ^abA^	−0.362 ± 0.4981 ^a^	0.027	0.033/0.092/0.412

Data are presented as mean ± SD (*n* = 16 piglets per group). Different superscripts (a, b) within a row indicate significant differences among groups (one-way ANOVA, *p* < 0.05, followed by Tukey’s post-hoc test). Superscripts A and B indicate a relative advantage or disadvantage compared with the control group, respectively. L/Q/C = Linear/Quadratic/Cubic. Polynomial regression analysis was performed to test for linear, quadratic, and cubi dose-response effects of *G. alkanivorans* on this parameter. It indicates a significant difference compared with the control group (*p* < 0.01). A, B: advantage or disadvantage vs. control. A higher F/B ratio correspond to a lower level of intestinal health.

## Data Availability

The original contributions presented in this study are included in the article. Further inquiries can be directed to the corresponding author(s).

## Abbreviations

ADG	Average Daily Gain
ADFI	Average Daily Feed Intake
ANOVA	Analysis of Variance
CC_50_	Half Cytotoxic Concentration
CFU	Colony-Forming Unit
CSFV	Classical Swine Fever Virus
F/B Ratio	Firmicutes/Bacteroidetes Ratio
FCR	Feed Conversion Ratio
IC_50_	Half-Maximal Inhibitory Concentration
IL	Interleukin
NF-κB	Nuclear Factor-Kappa B
OTU	Operational Taxonomic Unit
PAM	Porcine Alveolar Macrophage
PBS	Phosphate-Buffered Saline
PRV	Pseudorabies Virus
PRRSV	Porcine Reproductive and Respiratory Syndrome Virus
SCFA	Short-Chain Fatty Acid
TLR	Toll-Like Receptor
TNF-α	Tumor Necrosis Factor-α
TCID_50_	50% Tissue Culture Infectious Dose
qPCR	Quantitative Polymerase Chain Reaction
ΔΔCt	Comparative Threshold Cycle

## References

[1] Pan R., He Y., Yuan J., Zhao S., Ma M., Chai Z., Ji X., Hu X., He C., Zhou D. (2024). The role of antibiotic exposure as risk factor for IBD epidemic: An updated meta-analysis. J. Gastroenterol. Hepatol..

[2] Liu M., Luan H., Qiu W., Zhang Y., Feng W., Xu W., Wang F., Xuan H., Song P. (2025). Antibiotic alternatives in livestock feeding. Sci. Total Environ..

[3] Hong M.G., Song E.J., Yoon H.J., Chung W.H., Seo H.Y., Kim D., Lee D., Seo J.G., Lee H., Kim S.I. (2025). Clade-specific extracellular vesicles from Akkermansia muciniphila mediate competitive colonization via direct inhibition and immune stimulation. Nat. Commun..

[4] Vito R., Conte C., Traina G. (2022). A Multi-Strain Probiotic Formulation Improves Intestinal Barrier Function by the Modulation of Tight and Adherent Junction Proteins. Cells.

[5] Ma Y., Zhong Y., Tang W., Valencak T.G., Liu J., Deng Z., Mao J., Liu D., Wang S., Wang Y. (2025). *Lactobacillus reuteri* ZJ617 attenuates metabolic syndrome via microbiota-derived spermidine. Nat. Commun..

[6] Xing Y.Y. (2023). Application of Microecological Preparations in Animal Husbandry. Livest. Vet. Sci. Technol. Inf..

[7] Ministry of Agriculture and Rural Affairs of the People’s Republic of China (2025). Announcement No. 862: Catalogue of Feed Additive Varieties.

[8] Gu Y.L., Zhang X.X., Wei H.L., Yuan Z.Y., Gu J.G., Li S.G., Luo H.Y., Yao B. (2025). Thoughts on the Conservation and Utilization of Agricultural Microbial Germplasm Resources in China. Chin. J. Eng. Sci..

[9] Koga Y. (2022). Microbiota in the stomach and application of probiotics to gastroduodenal diseases. World J. Gastroenterol..

[10] Dianawati D., Mishra V., Shah N.P. (2016). Viability, Acid and Bile Tolerance of Spray Dried Probiotic Bacteria and Some Commercial Probiotic Supplement Products Kept at Room Temperature. J. Food Sci..

[11] Tavares J., Paixão S.M., Silva T.P., Alves L. (2024). New Insights on *Gordonia alkanivorans* Strain 1B Surface-Active Biomolecules: Gordofactin Properties. Molecules.

[12] Ioannou A., Berkhout M.D., Geerlings S.Y., Belzer C. (2025). Akkermansia muciniphila: Biology, microbial ecology, host interactions and therapeutic potential. Nat. Rev. Microbiol..

[13] Nahurira R., Ren L., Song J., Jia Y., Wang J., Fan S., Wang H., Yan Y. (2017). Degradation of Di (2-Ethylhexyl) Phthalate by a Novel *Gordonia alkanivorans* Strain YC-RL2. Curr. Microbiol..

[14] Yang Y., Zhang W., Zhang Z., Yang T., Xu Z., Zhang C., Guo B., Lu W. (2023). Efficient Bioremediation of Petroleum-Contaminated Soil by Immobilized Bacterial Agent of *Gordonia alkanivorans* W33. Bioengineering.

[15] Chen H., Li F., Lai W., Fang Y., Jiang M., Duan D., Yang X. (2021). cGAS/STING signaling pathways induces the secretion of typeIinterferon in porcine alveolar macrophages infected with porcine circovirus type 2. Chin. J. Biotechnol..

[16] Wang G., Li J., Liu Y. (2024). Integrated analysis of differential gene expression profiles in porcine alveolar macrophages induced by *Mycoplasma hyppneumoniae* strain 232. Pol. J. Vet. Sci..

[17] Casey A.M., Ryan D.G., Prag H.A., Chowdhury S.R., Marques E., Turner K., Gruszczyk A.V., Yang M., Wolf D.M., Miljkovic J.L. (2025). Pro-inflammatory macrophages produce mitochondria-derived superoxide by reverse electron transport at complex I that regulates IL-1β release during NLRP3 inflammasome activation. Nat. Metab..

[18] Sun Z. (2023). Differences in Inflammatory Responses and Proteomic Studies of PRRSV Infection in PAMs and PIMs. Ph.D. Thesis.

[19] Wang H., Xu Y., Feng W. (2021). Porcine Reproductive and Respiratory Syndrome Virus: Immune Escape and Application of Reverse Genetics in Attenuated Live Vaccine Development. Vaccines.

[20] Hu W., Tang D., Zeng Z., Wang B., Zhou M., Mao Y., Zhou P., He S. (2025). Research progress on the molecular mechanism of immune escape of porcine reproductive and respiratory syndrome virus. Virology.

[21] Rowland R.R.R., Brandariz-Nuñez A. (2024). Role of CD163 in PRRSV infection. Virology.

[22] Li P., Yang Z., Ma S., Hu G., Dong H., Zhang T. (2020). Susceptibility of porcine pulmonary microvascular endothelial cells to porcine reproductive and respiratory syndrome virus. J. Vet. Med. Sci..

[23] Zhao S.-S., Qian Q., Chen X.-X., Lu Q., Xing G., Qiao S., Li R., Zhang G. (2024). Porcine reproductive and respiratory syndrome virus triggers Golgi apparatus fragmentation- mediated autophagy to facilitate viral self-replication. J. Virol..

[24] An T.Q., Li J.N., Su C.M., Yoo D. (2020). Molecular and Cellular Mechanisms for PRRSV Pathogenesis and Host Response to Infection. Virus Res..

[25] Schifano E., Cicalini I., Pieragostino D., Heipieper H.J., Del Boccio P., Uccelletti D. (2020). In vitro and in vivo lipidomics as a tool for probiotics evaluation. Appl. Microbiol. Biotechnol..

[26] Chateau A., Schneewind O., Missiakas D. (2019). Extraction and Purification of Wall-Bound Polymers of Gram-Positive Bacteria. Methods Mol Biol..

[27] Hu J.B. (2023). Study on the Mechanism of Allicin Inhibiting Porcine Reproductive and Respiratory Syndrome Virus Infection. Master’s Thesis.

[28] Zhang X., Hartmann P. (2023). How to calculate sample size in animal and human studies. Front. Med..

[29] Chinese Journal of Tissue Engineering Research Editorial Office (2026). Reasonable estimation of sample size in animal experiments. Vet. Sci..

[30] Long Z. (2023). Study on the Effect of FADS1 Knockdown in Promoting Macrophage Phagocytosis of *Escherichia coli*. Ph.D. Thesis.

[31] Vivier E., Raulet D.H., Moretta A., Caligiuri M.A., Zitvogel L., Lanier L.L., Yokoyama W.M., Ugolini S. (2011). Innate or adaptive immunity? The example of natural killer cells. Science.

[32] Zhao T., Yue H., Peng J., Nie Y., Wu L., Li T., Niu W., Li C., Zhang Z., Li M. (2023). Degradation of xylan by human gut Bacteroides xylanisolvens XB1A. Carbohydr. Polym..

[33] Todorov S.D., Botes M., Guigas C., Schillinger U., Wiid I., Wachsman M.B., Holzapfel W.H., Dicks L.M. (2008). Boza, a natural source of probiotic lactic acid bacteria. J. Appl. Microbiol..

[34] Padilla M.A., Rodrigues R.A., Bastos J.C., Martini M.C., Barnabé A.C., Kohn L.K., Uetanabaro A.P., Bomfim G.F., Afonso R.S., Fantinatti-Garboggini F. (2015). Actinobacteria from Termite Mounds Show Antiviral Activity against Bovine Viral Diarrhea Virus, a Surrogate Model for Hepatitis C Virus. *Evid.-Based Complement*. Altern. Med..

[35] Noh M., Cho Y.S., Choi J., Song S.H., Cho J.Y., Vaidya B., Kim D. (2024). Enhanced anti-influenza activity of fermented yellow soybean extract and daidzein co-treatment on MDCK cells. Food Sci. Biotechnol..

[36] Park O.J., Kim J., Yang J., Yun C.H., Han S.H. (2019). Muramyl Dipeptide, a Shared Structural Motif of Peptidoglycans, Is a Novel Inducer of Bone Formation Through Induction of Runx2. J. Bone Miner. Res..

[37] Gluschko A., Farid A., Herb M., Grumme D., Krönke M., Schramm M. (2022). Macrophages target Listeria monocytogenes by two discrete non-canonical autophagy pathways. Autophagy.

[38] Wang H. (2019). The role of Erbin protein in autophagy induced by muramyl dipeptide in macrophages. Ph.D. Thesis.

[39] Ye W. (2013). Development of Oral Liquid of Mycobacterium Phlei and Its Clinical Application in Weaned Piglets. Master’s Thesis.

[40] Pupa P., Apiwatsiri P., Sirichokchatchawan W., Pirarat N., Nedumpun T., Hampson D.J., Muangsin N., Prapasarakul N. (2022). Microencapsulated probiotic *Lactiplantibacillus plantarum* and/or *Pediococcus acidilactici* strains ameliorate diarrhoea in piglets challenged with enterotoxigenic *Escherichia coli*. Sci. Rep..

[41] Kong J.M. (2021). Effects of Enzyme and Microecological Preparation Composite Agents on the Growth Performance, Intestinal Flora and Intestinal Mucosal Barrier Function of Weaned Piglets. Master’s Thesis.

[42] Ma S.M., Mao Q., Yi L., Zhao M.Q., Chen J.D. (2019). Apoptosis, Autophagy, and Pyroptosis: Immune Escape Strategies for Persistent Infection and Pathogenesis of Classical Swine Fever Virus. Pathogens.

[43] Deng J., Wu Z., Liu J., Ji Q., Ju C. (2022). The Role of Latency-Associated Transcripts in the Latent Infection of Pseudorabies Virus. Viruses.

[44] Zhang C. (2012). Study on the Effects of Complex Probiotics on the Growth Rate and Classical Swine Fever Antibody Level of Weaned Piglets. Master’s Thesis.

[45] Xie D., Li J., Luo P. (2020). Effects of Compound Microecological Preparation on Growth Performance, Diarrhea Rate, Cecum and Fecal Microorganism, Immune Function of Weaned Piglets. J. Domest. Anim. Ecol..

[46] Ukita M., Kuwata K., Tanaka E., Matsuyama R., Isoda N., Sakoda Y., Yamamoto T., Makita K. (2023). Exploring Appropriate Strategies for Vaccination against Classical Swine Fever under a Dynamic Change in Antibody Titer in Sows after Starting Vaccination in a Japanese Farm Setting. Transbound. Emerg. Dis..

[47] Zheng H.H., Fu P.F., Chen H.Y., Wang Z.Y. (2022). *Pseudorabies Virus*: From Pathogenesis to Prevention Strategies. Viruses.

[48] Tu W. (2012). Application Effect and Mechanism of Mycobacterium Phlei Preparation in Weaned Piglets’ Diet. Master’s Thesis.

[49] Liu Y., Gu W., Liu X., Zou Y., Wu Y., Xu Y., Han D., Wang J., Zhao J. (2022). Joint Application of *Lactobacillus plantarum* and *Bacillus subtilis* Improves Growth Performance, Immune Function and Intestinal Integrity in Weaned Piglets. Vet. Sci..

[50] Zamora-Pineda J., Kalinina O., Sperling A.I., Knight K.L. (2023). Mechanism of TLR4-Mediated Anti- Inflammatory Response Induced by Exopolysaccharide from the Probiotic Bacillus subtilis. J. Immunol..

[51] Bzówka M., Bagrowska W., Góra A. (2023). Recent Advances in Studying Toll- like Receptors with the Use of Computational Methods. J. Chem. Inf. Model..

[52] Mannochio R.H., Charron L.V., Van F.M. (2025). The microbiome diversifies long- to short-chain fatty acid-derived N-acyl lipids. Cell.

[53] Trinchieri G. (2003). Interleukin-12 and the regulation of innate resistance and adaptive immunity. Nat. Rev. Immunol..

[54] Huang Y., Tian C., Li Q., Xu Q. (2019). TET1 Knockdown Inhibits *Porphyromonas gingivalis* LPS/ IFN-γ-Induced M1 Macrophage Polarization through the NF-κB Pathway in THP-1 Cells. Int. J. Mol. Sci..

[55] Wang L., Hu S., Liu Q., Li Y., Xu L., Zhang Z., Cai X., He X. (2017). Porcine alveolar macrophage polarization is involved in inhibition of porcine reproductive and respiratory syndrome virus (PRRSV) replication. J. Vet. Med. Sci..

[56] Shi F., Xu Z., Gao P., Qu Y., Ge X., Zhang Y., Han J., Guo X., Zhou L., Yang H. (2025). African swine fever virus infection enhances CD14-dependent phagocytosis of porcine alveolar macrophages to promote bacterial uptake and apoptotic body-mediated viral transmission. J. Virol..

[57] Zeng W.Y., Xiao L., Liu J.Y., Han W.J., Wang Q., Liu Y.L., Fan Y., Xiao X., Wang Z.L. (2024). Regulatory mechanism of glycyrrhiza polysaccharide on intestinal damage and intestinal flora in enterotoxigenic Escherichia coli-induced weaning diarrhea piglets. Chin. Jourmnal Vet. Sci..

[58] Li X.Y. (2022). Study on the Protective Effects and Mechanisms of Short-Chain Fatty Acids on Severe Acute Pancreatitis. Master’s Thesis.

[59] Magne F., Gotteland M., Gauthier L., Zazueta A., Pesoa S., Navarrete P., Balamurugan R. (2020). The Firmicutes/Bacteroidetes Ratio: A Relevant Marker of Gut Dysbiosis in Obese Patients?. Nutrients.

[60] Li Y., Zhang H., Wang J. (2025). Dietary Supplementation with Gut commensal Lactobacillus Modulates Firmicutes/Bacteroidetes Ratio and Improves Intestinal Barrier Function in Weaned Piglets. J. Animal Sci. Biotechnol..

[61] Song L.L. (2023). Effects of Astragalus Polysaccharide on the Growth Performance, Antioxidant Capacity, Lipid Metabolism, and Gut Microbiota of Finishing Pigs. Master’s Thesis.

[62] Tian Z., Wang X., Duan Y., Zhao Y., Zhang W., Azad M.A.K., Wang Z., Blachier F., Kong X. (2021). Dietary Supplementation with Bacillus subtilis Promotes Growth and Gut Health of Weaned Piglets. Front. Vet. Sci..

